# Supplementary material for: ‘Microincisional trabeculectomy for glaucoma”
Source: PLoS One. 2023 May 19;18(5):e0286020. doi: 10.1371/journal.pone.0286020 (PMC10198474; doi:10.1371/journal.pone.0286020)
Supplement: S1 Table — (DOCX) [file pone.0286020.s001.docx]

Table S1: Comparison of clinical profile of patients undergoing either MIT alone or combined with cataract surgery

| **Variables** | **MIT alone (N=23)**  Mean ± standard deviation  or median(range) | **MIT+ cataract surgery (N=9)**  Mean ± standard deviation  or median(range) |
| --- | --- | --- |
| Age (years) | 56 (23-78) | 64(58-74) |
| Male:Female | 17:3 | 8:4 |
| Preop VA (decimals) | 0.2±0.2 | 0.6±0.5 |
| IOP at the time of surgery (mm Hg) | 21±11.1 | 23±12.05 |
| Mean deviation (dB) | -16±10.4 | -19±11.2 |

VA-visual acuity; IOP- intraocular pressure.
