# Supplementary material for: ‘Microincisional trabeculectomy for glaucoma”
Source: PLoS One. 2023 May 19;18(5):e0286020. doi: 10.1371/journal.pone.0286020 (PMC10198474; doi:10.1371/journal.pone.0286020)
Supplement: S2 Table — (DOCX) [file pone.0286020.s002.docx]

Table S2: Comparison of IOP outcomes and complications rates between eyes that underwent MIT alone or combined with cataract surgery

| **Variables** | **Mean ± standard deviation or N**  **N=23** | **Mean ± standard deviation or N**  **N=9** |
| --- | --- | --- |
| IOP week 1 (mm Hg) | 12±2.3 | 13±5.1 |
| IOP 1month (mm Hg) | 15±5.7 | 13±1.9 |
| IOP 6months (mm Hg) | 14±9.6 | 16±4.8 |
| Number of eyes requiring medication for IOP control  2 medications  1 medication | 4  1  3 | 0  0  0 |
| Hyphema | 3 | 1 |
| Number of eyes having transient IOP spikes | 4 | 1 |
| Repeat procedures/surgery | 1 | 0 |
| Hypereflective membrane on ASOCT | 2 | 3 |
| Loss of vision | 0 | 0 |

IOP-intraocular pressure; ASOCT- Anterior segment Optical Coherence Tomography
