# Supplementary material for: ‘Microincisional trabeculectomy for glaucoma”
Source: PLoS One. 2023 May 19;18(5):e0286020. doi: 10.1371/journal.pone.0286020 (PMC10198474; doi:10.1371/journal.pone.0286020)
Supplement: Attachment — Submitted filename: Comments plos one.docx [file pone.0286020.s004.docx]

Reviewer/editor comments-

Additional Editor Comments:
The authors need to be commended for this novel idea. However, the study suffers from a major limitation of follow up. They followed the patients and compared the results only till 2 months of follow-up, which is very short period, if we are looking at success of glaucoma surgeries. A follow-up of at least 6 months is desirable, to conclude if one technique is comparable in terms of IOP control and complications rate compared to other technique. It would add value to the study, to increase the follow-up of the patients, and re-analyze the results.

Answer: We thank the editorial team for suggesting this which is very appropriate. Therefore, we did follow-up patients for 6 months and included only those who completed at-least 6 months follow-up. We have now re-done the analysis based on patients that were included, with results also being represented in the new cohort selected.
